# A pilot study of alternative TrkAIII splicing in Merkel cell carcinoma: a potential oncogenic mechanism and novel therapeutic target

**DOI:** 10.1186/s13046-019-1425-3

**Published:** 2019-10-22

**Authors:** Lucia Cappabianca, Stefano Guadagni, Rita Maccarone, Michela Sebastiano, Alessandro Chiominto, Antonietta Rosella Farina, Andrew Reay Mackay

**Affiliations:** 1 Department of Applied Clinical and Biotechnological Sciences, University of L’Aquila, 67100 L’Aquila, Italy; 2 Department of Pathology, St. Salvatore Hospital, 67100 L’Aquila, Italy

**Keywords:** Merkel cell carcinoma, Merkel cell polyomavirus, MCPyV large T-antigen, Alternative TrkAIII splicing, Oncogenic activation mechanism, Therapeutic target

## Abstract

**Background:**

Merkel cell carcinomas (MCCs) are rare, aggressive, therapeutically-challenging skin tumours that are increasing in incidence and have poor survival rates. The majority are caused by genomic Merkel cell polyomavirus (MCPyV) integration and MCPyV T-antigen expression. Recently, a potential oncogenic role for the tropomyosin-related tyrosine kinase A receptor (TrkA) has been proposed in MCC. Alternative TrkAIII splicing is a TrkA oncogenic activation mechanism that can be promoted by SV40 large T-antigen, an analogue of MCPyV large T-antigen. In this pilot study, therefore, we have evaluated TrkAIII splicing as a novel potential oncogenic mechanism and therapeutic target in MCPyV positive MCC.

**Methods:**

Formalin-fixed paraffin-embedded MCC tissues, consisting of 10 stage IV, 1 stage IIIB, 1 stage IIB, 4 stage IIA and 2 stage I tumours, from patients diagnosed and treated from September 2006 to March, 2019, at the University of L’Aquila, L’Aquila, Italy, were compared to 3 primary basal cell carcinomas (BCCs), 3 primary squamous cell carcinomas (SCCs) and 2 normal skin samples by RT-PCR for MCPyV large T-antigen, small T-antigen, VP-1 expression and alternative TrkAIII splicing and by indirect IF for evidence of intracellular TrkA isoform expression and activation.

**Results:**

9 of 10 Recurrent stage IV MCCs were from patients (P.1–3) treated with surgery plus loco-regional Melphalan chemotherapy and remaining MMCs, including 1 stage IV tumour, were from patients treated with surgery alone (P. 4–11). All MCPyV positive MCCs exhibiting MCPyV large T-antigen expression (17 of 18MCCs, 90%) exhibited alternative TrkAIII mRNA splicing (100%), which was exclusive in a significant number and predominant (> 50%) in all stage IV MCCs and the majority of stage 1-III MCCs. MCCs with higher TrkAIII to 18S rRNA expression ratios also exhibited strong or intermediate immunoreactivity to anti-TrkA antibodies, consistent with cytoplasmic TrkAIII expression and activation. In contrast, the MCPyV negative MCC, BCCs, SCCs and normal skin tissues all exhibited exclusive fully-spliced TrkA mRNA expression, associated with variable immunoreactivity for non-phosphorylated but not phosphorylated TrkA.

**Conclusions:**

MCPyV positive MCCs but not MCPyV negative MCC, BCCs and SCCs exhibit predominant alternative TrkAIII splicing, with evidence of intracellular TrkAIII activation. This establishes a new potential MCC subset, unveils a novel potential MCPyV oncogenic mechanism and identifies TrkAIII as a novel potential therapeutic target in MCPyV positive MCC.

## Background

Merkel cell carcinomas (MCCs) are rare, aggressive, genetically unstable cutaneous neuroendocrine tumours, predominant in the elderly and in patients with chronic lymphocytic leukaemia, AIDS or organ transplants. Tumours are characterized by high rates of local-relapse, metastasis and mortality, associated with an overall 5-year survival rate of 60%, 2-year survival-rate of 26% in advanced stage and are currently treated by surgery, chemotherapy, radiotherapy and novel immune checkpoint inhibitors in advanced stage, depending on patient status [1–8].

Approximately 80% of MCCs are caused by genomic integration of Merkel cell polyomavirus (MCPyV) and MCPyV T-antigen expression, with the remaining 20% of non-viral MCCs exhibiting significant differences in gene transcription [9–13]. Poor survival rates and increasing incidence, however, underpin the need for greater understanding of the molecular mechanisms involved in MCC pathogenesis and their translation into novel therapy.

Recently, detection of extensive neurotrophin receptor tropomyosin-related tyrosine kinase A receptor (TrkA) immunoreactivity in MCC tissues has prompted suggestions of an oncogenic role for TrkA in this tumour-type [14, 15]. TrkA oncogenes are activated and play significant roles in many human cancers, and cancers driven by TrkA oncogenes exhibit profound, long-lived responses to novel clinically approved Trk inhibitors, such as Larotrectinib [16–18], suggesting that TrkA-targeted therapy may have a place in the treatment of MCC.

Oncogenic TrkA activation is achieved by gene amplification, novel gene fusion, point mutation, deletion mutation or alternative TrkAIII splicing [18–21]. Predominant oncogenic alternative TrkAIII splicing, originally identified and associated with advanced-stage metastatic disease and post-therapeutic relapse in human neuroblastomas (NBs), has also been detected in a subset of EGFR and EGFRvIII negative stage IV glioblastomas and in metastatic melanoma. TrkAIII is characterized by in-frame *TrkA* exons 6, 7 and 9 skipping, omission of receptor extracellular domain N-glycosylation sites required for cell surface receptor localization and the extracellular IG-like D4 domain involved in ligand-binding and prevention of spontaneous ligand-independent receptor-activation. TrkAIII oncogenic activity, confirmed by its capacity to transform NIH3T3 cells and promote oncogenic behaviour in neuroblastoma models, results from: receptor re-localization to pre-Golgi membranes, centrosomes and mitochondria; regulated ligand-independent activation within COP1/ERGIC membranes; PI3K/Akt/NF-κB survival-signalling; induction of a survival adapted ER-stress response; increased SOD2 expression enhancing resistance to oxidative-stress and promotion of a more angiogenic cancer stem cell-like phenotype. Furthermore, mitochondrial TrkAIII is stress-activated and promotes a metabolic switch to aerobic glycolysis and TrkAIII at the centrosome phosphorylates polo kinase-4 and α-tubulin leading to centrosome amplification, chromosome instability and enhanced microtubule polymerization [19–25].

Alternative TrkAIII splicing also represents a development and hypoxia-regulated physiological mechanism in normal neural-related stem/progenitor cells, thymocytes and thymic epithelial cells but not in differentiated neurons. In cancer cells, hypoxia promotes alternative TrkAIII splicing in KCNR, SK-N-BE, SH-SY5Y and Neuro 2 neuroblastoma, Jurkat T cell leukaemia, PC12 pheochromocytoma and TT medullary thyroid cancer cells and is constitutively predominant in U251 glioblastoma cells, suggesting that physiological alternative TrkAIII splicing is conserved and subverted into stress-regulated or constitutive oncogenic mechanisms in different human cancers [19–25].

In search of alternative mechanism that promote alternative TrkAIII splicing, we recently reported that SV40 large T-antigen promotes alternative TrkAIII splicing in neuroblastoma cells, unveiling a novel potential SV40 oncogenic mechanism [25]. Therefore, considering the causative roles of MCPyV and MCPyV large T-antigen and the potential role of TrkA in MCC pathogenesis and progression, and the analogous nature of SV40 and MCPyV large T-antigens [26], we initiated a pilot study to determine whether alternative TrkAIII splicing may represent an oncogenic mechanism and potential therapeutic target in MCC.

## Materials and methods

### Aim, design and setting

The aim of this study was to evaluate alternative TrkAIII splicing as a potential oncogenic mechanism and novel target in MCPyV positive MCC. Due to the rare nature of this tumour type, experiments were performed on a limited number of 18 FFPE MCC tissues from 11 patients, 3 individual BCCs and 3 individual SCCs from patients diagnosed and treated at the University of L’Aquila, L’Aquila, Italy from 2006 to 2019 and 2 normal skin samples, using appropriate RT-PCR-based and immunofluorescent (IF) techniques.

### Characteristics of participants and materials

The 18 MCCs, 3 basal cell carcinomas (BCCs), 3 squamous cell carcinomas (SCCs) FFPE tissues were from a 17 patient cohort, comprised of 18 MCCs from 11 patients (7 females and 4 males, with a mean ± SD age of 72.06 ± 12.24 years), consisting of 4 sequential recurrent stage IV MCCs from patient 1 (P.1, i-iv) [27]; 3 contemporary recurrent stage IV MCCs from patient 2 (P.2, i-iii); 2 contemporary recurrent stage IV MCCs from patient 3 (P.3, i and ii); 1 primary stage I and 1 recurrent stage IV MCC from patient 4 (P.4, i and ii); 1 recurrent stage IIIB MCC from patient P.5; 4 stage IIA and 1 stage IIB primary MCCs from patients P.6-P.10; 1 primary stage 1 MCC from patient P.11; 3 primary stage 1 BCCs from patients P.12-P.14; 3 primary SCCs from patients P.15-P.17 and 2 normal skin samples (NS1 and NS2) (Tables 1 and 2). MCC diagnoses were confirmed by histopathological positivity for cytokeratin AE1/AE3 and CD56 and individual clinical data are presented in Table 1. Written consent was obtained from all patients and the study was approved by ASL (n.1) Ethics committee, Abruzzo, Italy [10/CE/2018: 19 July 2018 (n.1419)].

**Table 1:** Clinical Characteristics of the patient cohort

Characteristics	MCC (*N* = 18)	BCC (*N* = 3)	SCC (*N* = 3)
Sex			
Female	7	2	2
Male	4	1	1
mean (SD)[range] years			
Total	72.06 (12.24) [41–93]	79 (7.21) [73–87]	78.3 (4.04) [74–82]
Female	71.77 (12.93) [41–93]	82 (7.07) [77–87]	80.5 (2.12) [79–82]
Localization			
Head and/or Neck	2/18 (11%)	2/3 (66.6%)	3/3 (100%)
Trunk	1/18 (5.5%)	1/3 (33%)	
Extremities	15/18 (83%)		
MCPyV expression			
Positive	17/18 (94.4%)	1/3 (trace level T-ag)	
Negative	1/18 (5.5%)	2/3 (66.6%)	3/3 (100%)
Stage AJCC (2019)			
I	2/18 (11.1%)	3/3 (100%)	3/3 (100%)
IIA	4/18 (22.2%)		
IIB	1/18 (5.5%)		
IIIB	1/18 (5.5%)		
IV	10/18 (60%)		
Current Status			
Dead	9/11 (82%)		
Alive	2/11 (18%)	3/3 (100%)	3/3 (100%)

**Table 2:** Patient and tumor information including: tumour-type: Merkel cell carcinoma (MCC), basal cell carcinoma (BCC), squamous cell carcinoma (SCC) or normal skin (NS); sample date; disease stage; level of MCPyV large T-antigen mRNA expression: high (H), medium (M), low (L) or no expression (N); mean (SD) TrkA and TrkAIII percentage of total TrkA (TrkA + TrkAIII) RT-PCR levels; mean (SD) densitometric TrkA and TrkAIII ratios to 18S rRNA RT-PCR levels; anti-TrkA and Y490 phosphorylated TrkA (anti-pY490 TrkA) immunoreactivity in tissue samples: strong (S), medium (M), weak (W) or negative (N); patient therapy: surgery (S) and locoregional Melphalan chemotherapy (C)

Patient	Date of sample (m/y)	Stage	Large T-ag	%TrkA Mean (SD)	%TrkAIII Mean (SD)	Mean (SD) Ratio TrkA:18S rRNA	Mean (SD) Ratio TrkAIII:18S rRNA	IF anti-TrkA	IF anti-pY490 TrkA	Therapy
MCC										
1	06–2014 (i)	IV	H	1.88 (4.5)	98.12 (4.5)	0.016 (0.002)	0.834 (0.03)	S	S	S/C
	09–2014 (ii)	IV	H	23.4 (8.4)	76.6 (8.4)	0.036 (0.007)	0.12 (0.01	M	M	S/C
	12–2014 (iii)	IV	M/H	23.1 (6.5)	76.9 (6.5)	0.08 (0.001)	0.44 (0.02)	S	M	S/C
	08–2015 (iv)	IV	H	8.6 (5.4)	91.4 (5.4)	0.034 (0.001)	0.4 (0.01)	M	M	S/C
2	02–2008 (i)	IV	H	31.9 (4.5)	68.1 (4.5)	0.2 (0.01)	0.43 (0.02)	S	S	S/C
	02–2008 (ii)	IV	H	1.3 (4.2)	98.7 (4.2	0.003 (0.0001)	0.26 (0.02)	S	S	S/C
	02–2008 (iii)	IV	H	1.9 (6.1)	98.1 (6.1)	0.002 (0.0001)	0.2 (0.01)	M	S	S/C
3	08–2011 (i)	IV	M	0.4 (4.2)	99.6 (4.2)	0.003 (0.0002)	0.68 (0.03)	S	S	S/C
	08–2011 (ii)	IV	M/H	0.8 (8.4)	99.2 (8.4)	0.001 (0.0003)	0.09 (0.002)	M	W	S/C
4	10–2012 (i)	I	M/H	6.2 (8.4)	93.8 (8.4)	0.02 (0.0013)	0.75 (0.02)	S	S	S
	02–2013 (ii)	IV	M	12.4 (8.2)	87.6 (8.2)	0.002 (0.0001)	0.08 (0.002)	M	M	S
5	02–2017	IIA	H	16.8 (7.9)	83.2 (7.9)	0.14 (0.005)	0.72 (0.002)	S	W	S
6	09–2006	IIA	H	2.7 (8.4)	97.3 (8.4)	0.003 (0.0002)	0.09 (0.003)	L	N	S
7	01–2010	IIA	L/M	28.5 (6.8)	71.5 (6.8)	0.08 (0.002)	0.19 (0.003)	L	M	S
8	11–2007	IIB	M/H	58.5 (6.7)	41.5 (6.7)	0.2 (0.002)	0.18 (0.003)	S	M	S
9	01–2013	IIA	H	47.4 (6.5)	52.6 (6.5)	0.19 (0.003)	0.22 (0.004)	S	S	S
10	01–2006	IIIB	H	58.5 (6.7)	41.5 (6.7)	0.055 (0.001)	0.08 (0.003)	L	N	S
11	01–2019	I	N	98.6 (0.2)	1.4 (0.2)	0.4 (0.002)	0.008 (0.001)	L	N	S
BCC										
12	01–2018	I	N	99.5 (0.04)	0.5 (0.04)	0.1 (0.02)	0.004 (0.0001)	N	N	S
13	02–2018	I	N	99.8 (0.02)	0.2 (0.02)	0.04 (0.002)	0.08 (0.002)	W	N	S
14	01–2019	I	L	99.9 (0.04)	0.1 (0.04)	0.213 (0.012)	0.02 (0.001)	N	N	S
SCC										
15	02–2019	I	N	99.8 (0.02)	0.2 (0.02)	0.18 (0.002)	0.001 (0.0002)	N	N	S
16	01–2019	I	N	99.8 (0.05)	0.2 (0.05)	1.3 (0.03)	0.004 (0.0002)	S	N	S
17	03–2019	I	N	99.04 (0.05)	0.06 (0.05)	0.17 (0.02)	0.008 (0.0002)	W	N	S
Normal Skin										
NS 1			N	99.6 (0.02)	0.4 (0.02)	0.45 (0.01)	0.0001 (0.00002)	S	N	S
NS 2			N	99.3 (0.01)	0.7 (0.01)	0.66 (0.023)	0.0001 (0.00002)	S	N	S

### RNA extraction, RT-PCR and sequence analysis

RNAs were purified from 50 μm MCC, SCC and BCC FFPE sections, using a total RNA purification kit for FFPE tissues, as directed (Thermo Fisher Scientific, CA), RNA purity and concentration was assessed using a nanodrop spectrophotometer, as directed (Thermo Fisher Scientific, CA) and purified RNAs reverse transcribed using a reverse transcription kit, as directed (Thermo Fischer Scientific, CA). Reverse transcription reactions (RTs) were then subjected to PCR using the following gene-specific primers: TrkA exon 5/6 splice-junction: 5′-CTGCAGTGTCATGGGCAA-3′ and 5′-CACATCCACCGAGGCATT-3′; TrkAIII exon 5–8 (covering the TrkAIII 5/8 splice junction): 5′-CTGCAGTGTCATGGGCAA-3′ and 5′-ACCAGTGGTGCATCTCCAC-3′; TrkA exons 3–8: 5′-AGTGGTCTCCCGTTTCGTGGCGCCA and 5′-ACCAGTGGTGCATCTCCAC-3′; GAPDH: 5′-CTGCACCACCAACTGCTTAG-3′ and 5′-GCAGTGATGGCATGGACTGT-3′; 18S rRNA: 5′-AAACGGCTACCACATCCACG-3′ and 5′-CCTCGAAAGAGTCCAGTATTG-3′; MCPyV VP1: 5′-CAACGAAAATTTGCCAGCTTA-3′ and 5’TTTAACAGAATATTGCCTCCCAC-3′; MCPyV small T-antigen: 5′-TGCCACCAGTCAAAACTTTC-3′ and 5′-AGCAAAAAAACTGTCTGACGTG-3′, and MCPyV large T-antigen: 5′-AAGGACCCATACCCAGAGGAAG-3′ and 5′-CCAACTCAAGATCCAGAAAGCC-3′. Primer sets were designed to generate 100 bp products to mitigate problems of RNA fragmentation in FFPE tissues [28] and facilitate comparative densitometric analysis. Optimum reverse transcription reaction (RT) dilutions for PCR were established by serial dilution in order to generated products within the linear-range. For TrkA, TrkAIII, MCPyV VP1, MCPyV small T-antigen and large T-antigen, RTs were undiluted, diluted 1:100 for GAPDH and 1:1,000 for 18S rRNA. RT-PCRs were performed in duplicate and repeated for each sample (*n* = 4). For densitometric analysis, 100 bp TrkA, TrkAIII, GAPDH, 18S rRNA, MCPyV VP1, MCPyV small T-antigen and MCPyV large T-antigen RT-PCR products from individual tissue samples were compared within the same 1% agarose gels, digitally photographed and analysed using Image J software (ImageJ bundled with Java 1.8.0_172) [29]. Inter-gel comparisons were made using common 18S rRNA RT-PCR product and DNA ladder standards.

### Indirect immunofluorescence (IF)

FFPE 5 μm sections were de-paraffinized, re-hydrated and processed for antigen retrieval by incubation in 0.01 M sodium citrate buffer [pH 6.0] for 20 min at 98 °C. Sections were blocked in BSA/TX100 blocking solution, incubated overnight at 4 °C with polyclonal rabbit anti-human TrkA (C14, Santacruz, CA); polyclonal rabbit anti-human Y490-phopsphorylated TrkA (pY490-TrkA, Cell Signalling Technologies, CA) or mouse anti-human γ-tubulin (Santacruz, CA) primary antibodies, diluted 1:50 in blocking solution, washed then incubated with fluorochrome-conjugated secondary antibodies (Alexa Fluor, ThermoFisher Scientific, CA), diluted 1:1000 in blocking solution for 2 h at room temperature. Slides were then washed, mounted in VectaShield with DAPI (Vector Laboratories, CA) and visually scored with strong, intermediate, weak or negative immunoreactivity by scanning confocal microscopy (Leica TCS SP_5_ II).

### Statistical analysis

Densitometric data were analysed statistically by Student’s *t*-test, using the online *t*-test calculator at https://www.graphpad.com/quickcalcs/ttest1.cfm and statistical significances were associated with probabilities of ≤0.05.

## Results

### RT-PCR

**Fig. 1:**
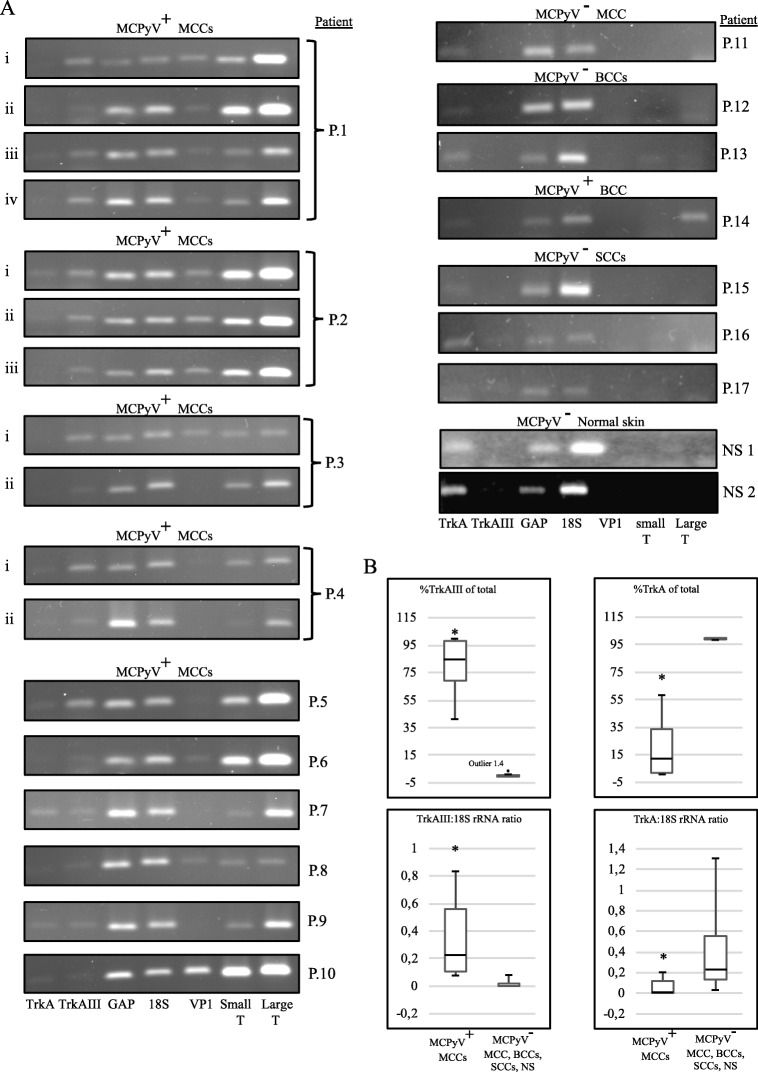
**a**) RT-PCRs demonstrating predominant alternative TrkAIII splicing over fully-spliced TrkA expression, compared to GAPDH (GAP), 18S rRNA (18S), MCPyV VP1, small T-antigen (Small T) and large T- antigen (Large T) RT-PCR products, in undiluted (TrkA, TrkAIII, VP1, Small T and Large T), 1:100 diluted (GAP) and 1:1000 diluted (18S) RT reactions of MCC RNAs (500 ng) (MCPyV^+^) compared to exclusive expression of fully spliced TrkA in RNAs (500 ng) from MCPyV negative (MCPyV^−^) MCC, BCCs, SCCs and normal skin samples, grouped by patient (P) number (10 ul loads per lane). **b** Box plots demonstrating significantly enhanced TrkAIII percentage of total TrkA (TrkA + TrkAIII) RT-PCR products and significantly reduced TrkA percentage of total (TrkA + TrkAIII) RT-PCR products (upper left and right box plots * p < 0.0001, df = 24), in MCPyV positive (MCPyV^+^) MCCs compared to MCPyV negative (MCPyV^−^) MCC, BCCs, SCCs and normal skin (NS) samples plus box plots demonstrating a significantly enhanced TrkAIII to 18S rRNA RT-PCR densitometric ratio in MCPyV positive (MCPyV^+^) MCCs compared to MCPyV negative (MCPyV^−^) MCC, BCCs, SCCs and normal skin (NS) samples (lower left box plot * =0.0009, df = 24) and a significantly reduced TrkA to 18S rRNA RT-PCR densitometric ratio in MCPyV positive (MCPyV^+^) MCCs compared to MCPyV negative (MCPyV^−^) MCC, BCCs, SCCs and normal skin (NS) samples (lower right box plot * =0.0015, df = 24)

**Fig. 2:**
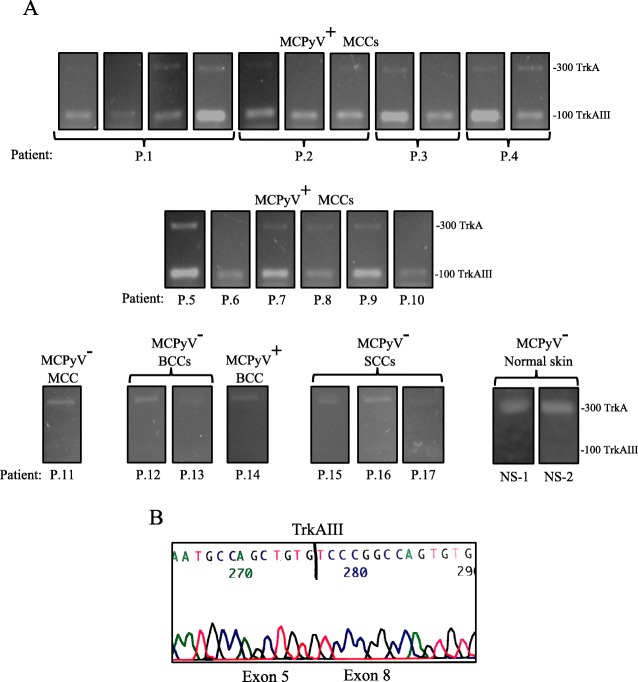
**a**) Representative RT-PCR reactions demonstrating predominant levels of 100 bp TrkAIII compared to 300 bp TrkA RT-PCR products in MCPyV positive (MCPyV^+^) MCCs grouped by individual patient (P) generated from TrkA exon 5–8 primers (Upper and middle RT-PCRs) plus exclusive 300 bp TrkA RT-PCR products generated from MCPyV negative (MCPyV^−^) MCC, BCCs, SCCs and normal skin samples, using the same primers. All reactions were performed using undiluted RT reactions from FFPE tissue RNAs (500 ng) (10 μl loads per lane). **b** Representative direct PCR sequence of the TrkAIII exon 5–8 splice junction in a RT-PCR fragment generated from a stage IV MCPyV positive MCC (patient 1 (i)), using the exon 3–8 primer set

RT-PCR detected MCPyV large T-antigen and small T-antigen expression in 17/18 (≈ 94%) MCCs, MCPyV VP-1 expression in 11/18 (≈ 61%) MCCs and trace level MCPyV large T-antigen expression in 1/3 BCCs. In contrast, MCPyV large T-antigen, small T-antigen and VP1 expression were not detected in 1 primary stage I MCC, 2/3 BCCs, 3/3 SCCs or 2 normal skin samples (Fig. 1a, Table 2). RT-PCR detected TrkAIII expression in all MCCs positive for MCPyV large T-antigen expression (100%), with predominant TrkAIII over fully spliced TrkA expression (> 50%) detected in the majority and exclusive TrkAIII mRNA expression in a significant number of MCCs. In contrast, MCPyV negative MCC, BCCs, SCCs and normal skin samples all exhibited exclusive expression of fully-spliced TrkA but not TrkAIII mRNA (Fig. 1a, Table 2). Densitometric analysis, revealed that MCPyV large T-antigen positive MCCs exhibited a significantly higher TrkAIII percentage of total TrkA expression corresponding to 80.92 ± 19.37% (mean ± SD) compared to 0.42 ± 0.397% in MCPyV negative MCC, BCCs, SCCs and normal skin (*p* < 0.0001, df = 24) (Fig. 1b and Table 2) that ranged from 76.6 ± 8.4% (*n* = 4) to 98.12 ± 4.5% (n = 4) in the 4 sequential recurrent stage IV MCCs (i-iv) from patient P.1; 68.1 ± 4.5% to 98.7 ± 6.1% in the 3 contemporary recurrent stage IV MCCs (i-iii) from patient P.2; 99.2 ± 8.4% (n = 4) to 99.6 ± 4.2% (n = 4) in the 2 contemporary stage IV recurrent MCCs (i and ii) from patient P.3; 87.6 ± 8.2% (n = 4) to 93.8 ± 8.6% (n = 4) in the primary stage I (i) and recurrent stage IV (ii) MCCs from patient P.4, and 41.5 ± 6.7% (n = 4) to 97.3 ± 6.4% (n = 4) in the remaining stage 1-III MCPyV positive MCCs from patients P.5–10 (Table 2). Consistent with this, MCPyV positive MCCs also exhibited a significantly lower 19.49 ± 19.71% (mean ± SD) TrkA percentage total TrkA expression compared to 99.48 ± 0.41% in MCPyV negative MCC, BCCs, SCCs and normal skin (*P* < 0.0001, df = 24) (Fig. 1b, Table 2). Furthermore, MCPyV positive MCCs exhibited a significantly lower TrkA:18S rRNA RT-PCR ratio of 0.063 ± 0.07 (mean ± SD) compared to 0.39 ± 0.37 in MCPyV negative MCC, BCCs, SCCs and normal skin (*p* = 0.0015, df = 24) and a significantly higher TrkAIII:18S rRNA ratio of 0.34 ± 0.25 (mean ± SD) compared to 0.014 ± 0.024 in MCPyV negative MCC, BCCs, SCCs and normal skin (*p* < 0.0009, df = 14) (Fig. 1b, Table 2). These results were also corroborated by comparing the 100 bp TrkAIII to the 300 bp TrkA RT-PCR product generated by the TrkA exon 5–8 primer set (Fig. 2a), despite the potential negative influence of RNA fragmentation in FFPE tissues on detection of fully spliced TrkA [28]. TrkAIII identity was confirmed in selected MCCs by direct PCR sequencing of the 300 bp fragment generated from the TrkA exon 3–8 primer set (Fig. 2b).

### Indirect IF

**Fig. 3:**
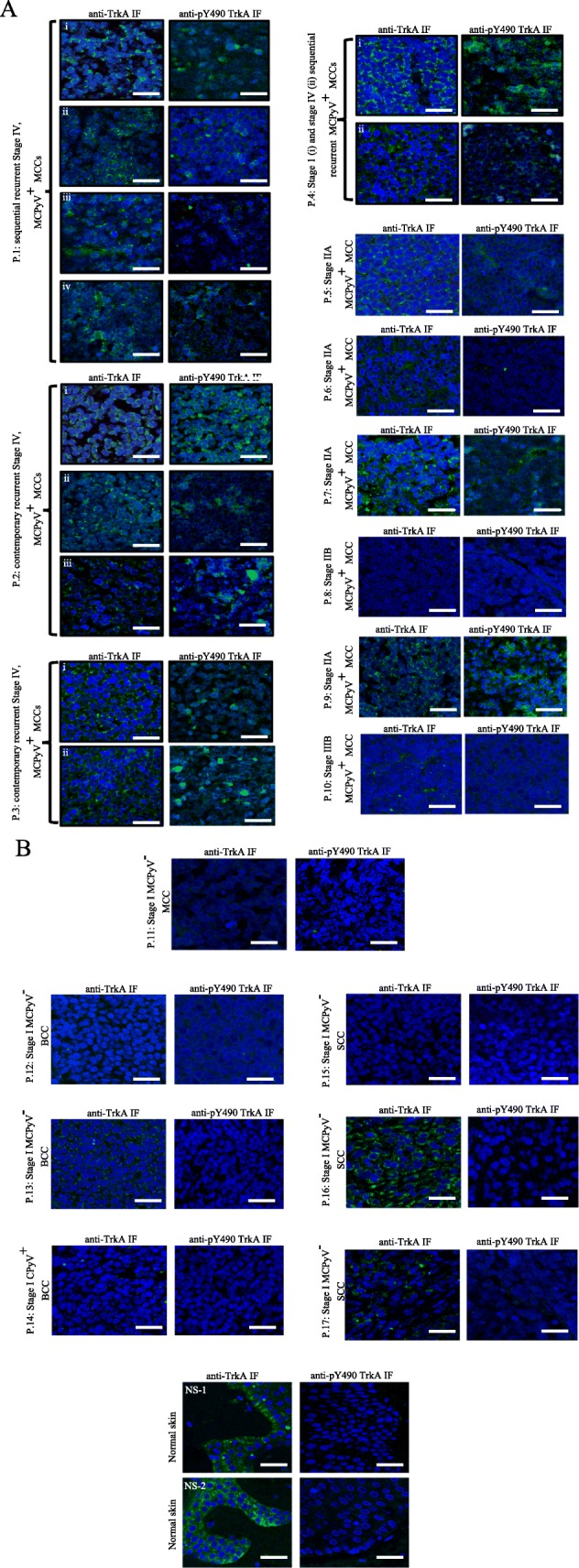
Indirect IF micrographs demonstrating nuclei (blue) and differences in IF immunoreactivity (green) to antibodies against TrkA (anti-TrkA) and Y490 phosphorylated TrkA (anti-pY490 TrkA) in: **a**) sequential recurrent stage IV MCCs (patients P.1 (i-iv), contemporary recurrent stage IV MCCs (patients P.2 (i-iii) and P4 (i)), and individual stage 1-IV MCPyV large T-antigen positive (MCPyV^+^) MCCs (patients P.4 (i) and P.5–10) and **b**) in an MCPyV negative (MCPyV^−^) MCC (patient P.11), 2 MCPyV negative BCCs (patients, P.12 and P.13), 1 MCPyV large T-antigen positive BCC (patient P.14), 3 MCPyV negative SCCs (patients P.15–17) and 2 MCPyV negative normal skin samples (NS1 and 2) (bar = 50 μm)

**Fig. 4:**
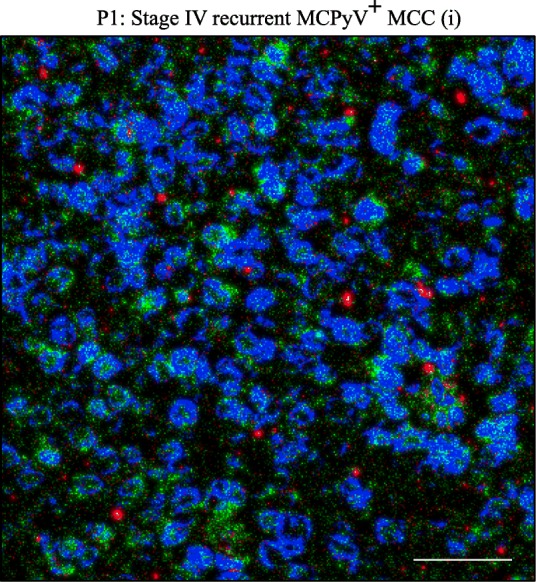
Indirect IF micrograph demonstrating the co-localisation (yellow) of TrkAIII (green) and γtubulin (red) in a stage IV, MCPyV positive (MCPyV^+^) MCC (Patient, P.1 (i)) (bar = 50 μm)

Using antibodies against TrkA and Y490 phosphorylated TrkA that also recognize TrkAIII [20], strong or intermediate immunoreactivity for both antibodies characterised MCPyV positive MCCs with high-level MCPyV large T-antigen expression (Fig. 3a), exclusive TrkAIII mRNA expression and relatively high TrkAIII to 18S rRNA RT-PCR ratios (Figs. 1a and 2a and Table 2), whereas weak immunoreactivity characterized MCPyV large T-antigen positive MCCs exhibiting low TrkAIII to 18S rRNA ratios (Figs. 1a, 2a and 3a and Table 2). The MCPyV negative stage I MCC (patient P.11) was negative for both TrkA and phosphorylated TrkA immunoreactivity (Fig. 3b, Table 2), despite low-level exclusive fully-spliced TrkA mRNA expression (Figs. 1a and 2a and Table 2). All recurrent stage IV MCCs (patients P.1–3) exhibited strong or intermediated immunoreactivity to both antibodies (Fig. 3a and Table 2). Strong TrkA immunoreactivity characterized normal skin epithelia exhibiting exclusive fully spliced TrkA mRNA expression and high TrkA:18S rRNA ratios, and also characterized the tumour component of 1 SCC, exhibiting exclusive TrkA mRNA expression. In contrast, scant immunoreactivity characterized 1/3 BCCs and 1/3 SCCs and the remaining BCCs (2/3) and SCC (1/3) were negative for TrkA immunoreactivity (Fig. 3b and Table 2). Immunoreactivity for phosphorylated TrkA was not detected in any of the MCPyV negative MCC, BCCs, SCCs or normal skin samples (Fig. 3b and Table 2). These data provide evidence for intracellular TrkAIII expression and activation in MCPyV positive MCCs and in particular stage IV tumours exhibiting exclusive TrkAIII mRNA expression, whereas fully spliced inactive TrkA expression characterised normal skin epithelia and the tumour component of some but not all MCPyV negative BCCs and SCCs. TrkA immunoreactivity also co-localized with centrosome γ-tubulin in a stage IV MCPyV positive MCC exhibiting exclusive TrkAIII mRNA expression (Fig. 4, Patient P. 1 i).

## Discussion

In this pilot study of alternative TrkAIII splicing in MCC, we report that MCPyV, presumably through large T-antigen, promotes alternative TrkAIII mRNA splicing in MCPyV positive tumours, with evidence of both intracellular TrkAIII expression and activation. We propose that this represents a novel potential MCPyV oncogenic mechanism, may establish a new MCPyV positive MCC subtype and identifies TrkAIII as potential target that may drive new therapeutic strategies.

MCPyV large T-antigen expression was detected in ≈90% of MCCs tissues, adding to reports that genomic MCPyV integration and MCPyV large T-antigen expression cause ≈80% of MCCs [9–13]. Trace-level MCPyV large T-antigen was also detected in a BCC, suggesting that MCPyV may also be involved in non-MCC cutaneous pathology. Although this supports reports of MCPyV in BCC tissues [30, 31], MCPyV also forms part of the normal cutaneous microbiome and does not integrate into the genomes of non-MCC carcinomas, suggesting a coincidental rather than causative association with BCC [30–32].

All MCPyV positive MCCs exhibiting MCPyV large T-antigen mRNA expression also exhibited alternative TrkAIII splicing that did not fall below 40% of total TrkA expression, by densitometric RT-PCR analysis, in this MCC cohort. Furthermore, alternative TrkAIII splicing predominated (> 50%) over that of fully spliced TrkA in the majority and was exclusive in a significant number of MCPyV positive MCCs, confirming a close relationship between MCPyV infection, MCPyV large T-antigen expression and alternative TrkAIII splicing in MCC. In contrast, the MCPyV negative MCC, BCCs, SCCs and normal skin samples all exhibited exclusive expression of fully spliced TrkA mRNA. These observations extend our previous report that alternative TrkAIII splicing is promoted by SV40 large T-antigen in neuroblastoma cells, by identifying a close relationship between MCPyV large T-antigen expression and alternative TrkAIII splicing in MCC, consistent with the analogous nature of SV40 and MCPyV large T-antigens [26], and identifies alternative TrkAIII splicing as a novel potential MCPyV oncogenic mechanism. How polyomavirus large T-antigens promote alternative TrkAIII splicing remains to be elucidated but is likely to involve altered RNA polymerase elongation rates, previously implicated in SV40 T-antigen induced alternative splicing [33, 34].

All stage IV MCPyV positive MCCs exhibited predominant TrkAIII mRNA expression that was exclusive in a significant number, confirming that predominant TrkAIII mRNA expression associates with advanced stage MCPyV positive MCC. Furthermore, recurrent stage IV MCCs following Melphalan loco-regional chemotherapy [27] continued to exhibit MCPyV large T-antigen and predominant TrkAIII expression, indicating that Melphalan chemotherapy did not modify the relationship between MCPyV large T-antigen and alternative TrkAIII splicing in recurrent tumours. Variations in the TrkAIII expression ratio to 18S rRNA, however, indicates that this relationship does not extend to MCPyV promotion of *TrkA* transcription, which suggests that TrkAIII involvement in MCC would be restricted to tumors exhibiting constitutive *TrkA* transcription, unveils a novel potential oncogenic post-transcriptional function for MCPyV large T-antigen in addition to inhibition of tumour suppressor activity [26] and is consistent with reports linking Alk and EGFR receptor tyrosine kinase oncogenes to MCC [3, 35].

Although TrkAIII-specific antibodies are not available at present, the TrkA and Y490 phosphorylated TrkA antibodies used for IF have previously been shown to recognize both TrkA and TrkAIII and the anti-Y490 phosphorylated TrkA antibody shown to recognize NGF-activated TrkA and spontaneously active TrkAIII but not their inactive counterparts by IF [19–23]. Therefore, immunoreactivity to these antibodies detected in stage IV MCPyV positive MCCs exhibiting exclusive TrkAIII mRNA expression, with the highest levels detected in tissue with high TrkAIII to 18S rRNA expression ratios, is most likely to represent the intracellular expression, phosphorylation and activation of TrkAIII. However, we have been unable to confirm this by immunoprecipitation/Western blotting due to problems in obtaining sufficient quantities of purified proteins from the limited amount of IF positive FFPE MCC tissues available.

The lack of phosphorylated TrkA immunoreactivity in the MCPyV negative MCC and in BCC, SCC, and normal skin tissues exhibiting exclusive fully spliced TrkA mRNA expression and TrkA immunoreactivity confirms a marked difference in TrkA isoform phosphorylation status in MCPyV positive MCCs and MCPyV negative MCC, BCCs, SCCs and normal skin, and indicates that fully spliced TrkA in BCC, SCC and normal skin is either inactive or activated below the threshold of detection in FFPE tissues. In other cancers, immunohistochemical detection of TrkA phosphorylation has been shown to predict poor outcome and an aggressive phenotype in melanoma and to predict response to Larotrectinib therapy in cancers driven by Trk fusion oncogenes [16–18, 36], suggesting that the detection of TrkAIII expression and phosphorylation in MCPyV positive MCCs may eventually provide similar information of diagnostic and therapeutic significance.

Although the influence of TrkAIII on MCC behaviour remains to be elucidated, TrkAIII oncogenic activity, confirmed by its capacity to transform NIH3T3 cells and promote oncogenic behaviour in neuroblastoma models, has been reported to involve re-localization of this compromised receptor to pre-Golgi membranes, centrosomes and mitochondria. This results in regulated ligand-independent activation within intracellular COP1/ERGIC membranes, inducing: PI3K/Akt/NF-κB survival-signalling; a survival adapted ER-stress response; increased SOD2 expression enhancing resistance to oxidative-stress and a more angiogenic cancer stem cell-like phenotype. Furthermore, mitochondrial TrkAIII exhibits stress-induced calcium-dependent activation, phosphorylates mitochondrial pyruvate dehydrogenase kinase-1 and promotes a metabolic switch to aerobic glycolysis and at the centrosome TrkAIII phosphorylates α-tubulin and polo kinase-4, promoting microtubule polymerization, inducing centrosome amplification and increasing genetic instability [19–25]. The detection of predominant TrkAIII splicing in advanced stage and recurrent stage IV MCPyV positive MCCs also adds to reports that predominant TrkAIII splicing associates with advanced stage metastatic disease and post-therapeutic relapse in neuroblastoma, characterizes a subset of advanced stage EGFR and EGFRvIII negative glioblastomas and has been detected in metastatic melanoma [19–25], supporting the hypothesis that predominant alternative TrkAIII splicing and intracellular TrkAIII activation represents a novel oncogenic mechanism and potential target in a subset of MCPyV positive MCCs.

Predominant TrkAIII mRNA expression in advanced stage and recurrent MCPyV positive MCCs, with evidence of intracellular TrkAIII expression and activation, also extends previous reports of a potential oncogenic role for TrkA in MCC [14, 15] but would negate a proposed requirement for NGF-expressing MCC-infiltrating inflammatory cells for activation [15], which may be more relevant to MCPyV negative MCCs, BCCs and SCCs that express fully spliced TrkA receptors (this study, [37, 38]). In addition, confocal IF in an advanced stage MCPyV positive MCC exhibiting exclusive TrkAIII mRNA expression also detected anti-TrkA immunoreactivity co-localised with γ-tubulin, suggesting that TrkAIII may localise to the centrosome in MCCs. This is consistent with previous reports that TrkAIII binds γ-tubulin and localizes to the centrosome in neuroblastoma cells, causing centrosome amplification and genetic instability [21] and suggests that TrkAIII may also be involved in the centrosome amplification and genetic instability that characterises MCC [3].

In contrast to MCPyV positive MCCs, MCPyV negative MCC, BCCs, SCCs and normal skin samples exhibiting exclusive fully spliced TrkA mRNA expression, also exhibited variable TrkA immunoreactivity, strongest in normal skin epithelia and in 1 SCC but scant or non-existent in other BCC and SCC tissues, but were not immunoreactive for phosphorylated TrkA. This support reports of TrkA expression in normal skin epithelia and a minority of BCC and SCCs but indicates that TrkA is not activated in these tissues, suggesting a limit to TrkA involvement in BCC and SCC, despite their keratinocyte origins [39].

Predominant alternative TrkAIII mRNA splicing with evidence of intracellular TrkAIII activation, associated with MCPyV infection and MCPyV large T antigen expression in MCCs, may establish a new subtype and identifies TrkAIII as a potential target that could lead to novel therapeutic strategies. Within this context, potential inhibitory therapeutic strategies could include: siRNA inhibition of MCPyV large T antigen expression to prevent alternative TrkAIII splicing; siRNA and PNA inhibitors of TrkAIII expression, reported to enhance the sensitivity of TrkAIII expressing cancer cells to cytotoxic agents; TRAIL, reported to induce apoptosis in TrkAIII expressing neuroblastoma cells, and cell-permeable small molecule TrkA inhibitors, reported to inhibit TrkAIII activity and sensitize TrkAIII expressing cancer cells to cytotoxic agents [19–25, 40]. In this respect, the FDA-approved Trk inhibitor “Larotrectinib” is of particular therapeutic interest, as it inhibits the activity of fusion, mutation and deletion-activated TrkA oncogenes, exhibits remarkable durable efficacy in a wide range of advanced stage human cancers driven by TrkA oncogenes [16–18] and could be tested, as third line therapy, in this MCPyV positive TrkAIII expressing MCC subtype.

## Conclusions

In conclusion, this pilot study of rare MCC FFPE tissues clearly demonstrates that advanced stage MCPyV positive MCCs exhibit predominant, in some cases exclusive, alternative TrkAIII mRNA splicing with evidence of intracellular TrkAIII expression and activation potentially driven by MCPyV large T-antigen. We propose that this characterises and establishes a new MCPyV positive MCC-subtype, unveils a novel MCPyV oncogenic mechanism and potential oncogenic function for MCPyV large T-antigen, identifies TrkAIII as novel potential therapeutic target and provides a rational for the use of Trk inhibitors, such as Larotrectinib, in the treatment of this MCPyV positive TrkAIII expressing tumour subtype.

## Data Availability

The data sets used and/or analysed during this study are either included in this published article or are available from the corresponding author on reasonable request.

## Abbreviations

BCC: Basal cell carcinoma
DAPI: 4′,6′-diamidino-2-phenylindole
FFPE: Formaldehyde fixed paraffin-embedded
IF: Immunofluorescence
MCC: Merkel cell carcinoma
MCPyV: Merkel cell polyomavirus
RT-PCR: Reverse transcription polymerase chain reaction
SCC: Squamous cell carcinoma
TrkA: Tropomyosin-related kinase A
